# Advancing knowledge, maps and tools to address obesity and related socio-economic disparities in Europe: the OBCT project

**DOI:** 10.1080/16549716.2025.2517492

**Published:** 2025-07-18

**Authors:** Thao Minh Lam, Julie Aarestrup, Erica L. T. van den Akker, Jennifer L. Baker, Anna Banik, Bram J. Berntzen, Lucrezia Bertoni, Joline W. j. Beulens, Kirsten Bjørnsbo, Sheree Bryant, Luis Cereijo, Priyanka Choudhary, Valeria C. Cuenca Cuenca, Julia Diez, Ulf Ekelund, Majid Ezzati, Matthew O’Flynn, Manuel Franco, Jet van de Geest, Pedro Gullon, Lisa Heggie, Sondre H. Herstad, Søren Holm, Rachel Jackson Leach, Nanna Lien, Tim Lobstein, Aleksandra Luszczynska, Joreintje Mackenbach, Renate Meeusen, Paul Meijer, Biljana Meshkovska, Jouko Miettunen, Rozenn Nedelec, Jack Olney, Natalia Paduszyńska, Nishit Patel, Jaynaide Powis, Jayashankaramma S. Mohan Raju, Tessa Roseboom, Elisabeth F.C. van Rossum, Miguel A. Sanchez-Lastra, Fahmida Sarker, Franco Sassi, Sylvain Sebert, Maria Siwa, Josine Stuber, Jakob Tarp, Ulla Toft, Kamille A. B. Torp, Jorrit van Uhm, Roberto Valiente Borox, Alfred Wagtendonk, Maggie Wetzel, Euan Woodward, Jingmin Zhu, Jeroen Lakerveld, the OBCT consortium

**Affiliations:** a Department of Epidemiology and Data Science, Amsterdam UMC, Amsterdam, The Netherlands; b Amsterdam Public Health, Health Behaviors and Chronic Diseases, Amsterdam, The Netherlands; c Upstream Team, Amsterdam UMC, VU University Amsterdam, Amsterdam, The Netherlands; d Center for Clinical Research and Prevention, Copenhagen University Hospital - Bispebjerg and Frederiksberg, Copenhagen, Denmark; e Department of Pediatrics, Div Pediatric Endocrinology, Obesity Center CGG, Erasmus MC Sophia Children’s Hospital, Rotterdam, The Netherlands; f CARE-BEH Center for Applied Research on Health Behavior and Health, SWPS University, Wroclaw, Poland; g European Association for the Study of Obesity, Teddington, UK; h Public Health and Epidemiology Research Group, School of Medicine and Health Sciences, Universidad de Alcalá, Madrid, Spain; i Research Unit of Population Health, Faculty of Medicine, University of Oulu, Oulu, Finland; j Department of Sports Medicine, Norwegian School of Sports Sciences, Oslo, Norway; k Department of Chronic Diseases, Norwegian Institute of Public Health, Oslo, Norway; l Department of Epidemiology and Biostatistics, School of Public Health, Imperial College London, London, UK; m World Obesity Federation, London, UK; n Department of Nutrition, Faculty of Medicine, University of Oslo, Oslo, Norway; o Centre for Medical Ethics, HELSAM, University of Oslo, Oslo, Norway; p Julius Center for Health Sciences and Primary Care, University Medical Center Utrecht, Utrecht University, Utrecht, The Netherlands; q Medical Research Center Oulu, Oulu University Hospital and University of Oulu, Finland; r Department of Economics & Public Policy, Centre for Health Economics & Policy Innovation, Imperial College Business School, Imperial College London, London, UK; s Department of Internal Medicine, Division of Endocrinology, Obesity Center CGG, Erasmus MC, University Medical Center Rotterdam, Rotterdam, the Netherlands; t Department of Special Didactics, University of Vigo, Pontevedra, Spain; u Wellness and Movement Research Group (WellMove), Galicia Sur Health Research Institute (IIS Galicia Sur), SERGAS-UVIGO, Vigo, Spain; v Steno Diabetes Center, Steno Diabetes Center Copenhagen, Herlev, Denmark

**Keywords:** Maria Nilsson, Obesity, life-course, primary prevention, policy, lifestyle behaviours, socio-cultural factors, exposome, inequalities, co-creation, implementation

## Abstract

The steady rise in overweight and obesity in Europe disproportionately affects people and communities with a lower socio-economic position (SEP). Many obesity prevention approaches exist, but these have had limited reach and unsatisfactory effects thus far, especially in low-SEP populations. In this context, there is a need for implementation of effective individual-level and population-based preventive strategies that also tackle health inequalities. Effective strategies require consideration of the complex and cross-domain obesity risk factors across the life course. Feasible and acceptable strategies require multisectoral collaborations and innovative approaches, including a whole-of community and systems perspective. With the Horizon Europe-funded OBCT project, we aim to quantify the relative contribution of biological, socio-cultural and built environment factors to obesity and the interactions of these risks within and across various life course stages; and translate the resulting knowledge into practical, equitable, and effective tools for action. These tools will include: a comprehensive obesity risk screener; a map of the obesogenicity of neighbourhood environments as well as trends in obesity prevalence of each European country; recommendations for lifestyle behaviours (diet, physical activity, sedentary behaviours) to prevent obesity during key life transition stages; a decision support dashboard for policy makers; and co-developed toolboxes to support implementation of policy recommendations in low-SEP communities. OBCT’s outputs will highlight the areas and domains in which obesity should be targeted and will empower the research community, policymakers, health professionals and residents in Europe to adapt and implement strategies to effectively reduce obesity risk, particularly in low-SEP communities.

## Paper Context

- **Main findings**: Obesity has multifactorial causes across many domains, from genetic to social and environmental, which might not be equally distributed across the different population groups.
- **Added knowledge**: This design paper contributes to the methodological development of integrated risk models across these population groups using novel methods and rich, existing population health datasets across Europe.
- **Global health impact for policy and action**: This project aims to identify and stratify by population risk groups and design specific interventions with especially vulnerable groups in order to maximize public health impact across Europe.

## Background

**Figure 1.**
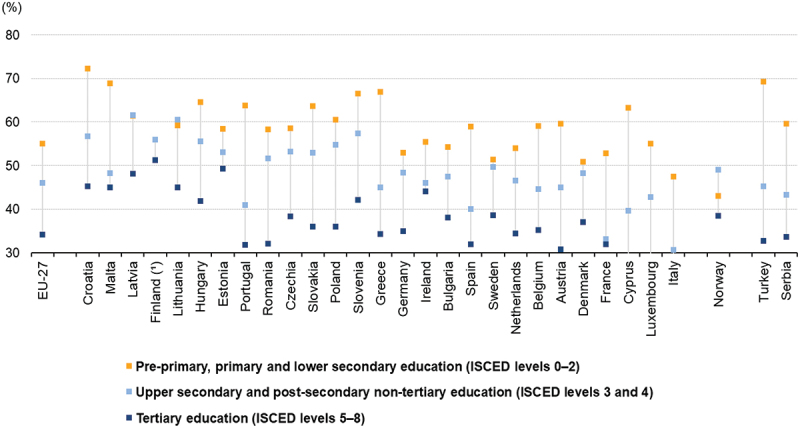
Inequalities in the population proportion (%) of overweight (including obesity) in women by educational level, 2019 (source: Eurostat [9]).

The prevalence of obesity has tripled in many European countries in the last decades [1]. Almost a quarter (23%) of adults in the European Region are now living with obesity [2]. Living with obesity increases the risk of a range of diseases [3], and strongly links to other emerging problems, such as mental health, the availability of healthy foods in a changing world (climate change, pollution), and socio-economic position (growing wealth gap, inflation, cost of living) [2]. Importantly, the obesity prevalence is not equally distributed. Obesity prevalence is particularly higher in individuals and communities that have a disadvantaged or lower socio-economic position (SEP) relative to others in their population. Furthermore, a clear social gradient can be seen whereby the prevalence of obesity in each SEP group is relatively higher than the next group above them in the social spectrum [4,5]. European societies are facing a critical period in which the COVID pandemic, inflation, and the energy crisis hit low-SEP populations particularly hard, further widening health inequalities between SEP groups [6–8]. The steepness of the overweight and obesity inequality gradient varies from country to country and those with lower levels of education (as a proxy for SEP) can be up to five times more likely to be affected by overweight and obesity in European adults (women in particular) than those with higher education (Figure 1). This in turn, predisposes their children and future generations towards health risks from early on, thereby transmitting obesity inequalities across generations [9,10].

The reasons for higher obesity rates in low-SEP communities are not fully clear, but likely due to a combination of factors including stress and the scarcity of resources that support healthier behaviours, such as financial means, health literacy, social support, access to a healthy neighbourhood environment, empowerment and access to obesity treatment (including medications) [10–14]. A deeper understanding of SEP-specific and life-course-specific risk factors and their interplay is needed. Gaining this new knowledge is essential, as the biological, socio-cultural and environmental drivers of obesity interact and their influence is very likely to change over the life course (i.e. the entirety of individual’s life from prenatal development onwards, covering life stages such as infancy and toddlerhood; early- and middle childhood; adolescence; early-, middle-, and late adulthood) [15,16]. Consequently, implementation of effective obesity prevention approaches also requires addressing of not only individual-level risk factors but also the broader socio-cultural and contextual influences that shape behaviours and health outcomes. Factors such as cultural norms, social support networks, and access to safe spaces for physical activity affect the feasibility and success of interventions.

Most of the current obesity-related policies, recommendations, and guidelines aiming to improve behavioural risk-factors of obesity are generic, for instance focusing on promoting healthy eating and physical activity of whole populations through national public health campaigns or food labelling [17–19]. A major limitation of such approaches is that they do not adequately account for the diversity of lived experiences or the structural inequities faced by under-resourced groups/individuals [20]. For example, many interventions fail to address barriers such as food insecurity, neighbourhood deprivation, and cultural influences on health behaviours. Existing individual-level interventions may be more tailored to individual needs, but generally have a limited reach and effects are rarely sustained over time [13,21–23]. Moreover, such interventions seem to be incapable of sustainably reducing socio-economic inequalities in obesity, especially in adults [24]. In terms of research, there is a need for greater attention, recognition, and awareness of communities with disadvantaged SEPs. Currently, the greatest gap in knowledge in the area of obesity risk is not regarding the number of risk factors, or their independent impact on risk, but rather how they interact with one another across the life course and which approaches may affect the critical risk factors with the aim of preventing obesity. In terms of outputs, there is a growing demand for evidence-based, actionable, and practical tools and measures that fill the gap between national policies and guidelines, and frontline practice in primary care and communities. To target intervention efforts, it is helpful to identify and map out the hotspots in Europe where obesity trends – especially the more harmful abdominal adiposity – increase.

To develop policies, interventions, and tools that can halt the rising prevalence of obesity, we need to understand the mechanisms that underlie the development of obesity, especially within subgroups at risk. A number of factors, such as overconsumption of energy-dense foods, a lack of physical activity, and prolonged sedentary behaviour increase the risk of developing obesity [25]. These behaviours are driven by a complex and dynamic interplay between biological, socio-cultural, and environmental risks [26]. The relative importance of these risks varies according to the life course stage and SEP of individuals [27–29]. To effectively improve behaviours related to a higher risk of obesity, tailored approaches are required to address the dynamics of the risk factors, across and between various life course stages. Therefore, we need to understand how and where these risk factors affect individuals and communities across the life course, how this relates to SEP, how risk factors can be identified in the individual to tailor interventions and which public policies effectively reduce these risks, and how these approaches can be implemented in the community. Primary prevention of obesity is essential, as the reversal of obesity is challenging and costly, even though there are new and more effective pharmacological interventions available; obesity-related non-communicable diseases (NCDs) impose significant costs for health and social care. In the current climate of austerity, prevention of obesity must be considered a priority [30], especially among those with a lower SEP whose access to care could be most restricted.

## Overall aim and objectives of the OBCT project

The overall aim of OBCT (Obesity: Biological, Socio-Cultural and Environmental Risk Trajectories) is to quantify biological, socio-cultural, and built environmental obesity risk factors and their interactions within and across various life stages and translate these findings into practical and effective tools to support policymaking. Our aspiration is to provide health professionals, policymakers, researchers, and the public with new knowledge, maps, and tools to support the sustainable prevention of obesity and the reduction of socio-economic inequalities therein.

This aim is broken down into three overarching goals, structured by *Knowledge*, *Maps,* and *Tools*, with ten underlying objectives:

KNOWLEDGE - *Extend the current understanding of obesity risks in various SEP communities across the life course*.

1. Assess biological, socio-cultural and environmental obesity risks and predictors, and the embedded role of SEP across the life course.
2. Determine the importance of specific behaviours to prevent obesity at key life stage transitions.
3. Develop and test a comprehensive obesity risk screener.

MAPS - *Map European obesity trends, obesogenic environments, and obesity-related cardio-metabolic disease risk*.

1. Provide country-specific estimates of trends in general and abdominal obesity at key stages of the life course across Europe.
2. Quantify obesogenic neighbourhood characteristics of all local administrative units within Europe.
3. Characterise obesity-related cardio-metabolic risk profiles over gender, age, SEP within representative European countries.

### TOOLS - provide tailored lifestyle recommendations and tools to implement policies as actions in the community

1. Develop recommendations for dietary behaviours, physical activity, and sedentary behaviours tailored to key life stages and socio-economic backgrounds.
2. Determine the potential impact of obesity-related policies on SEP-related inequalities in obesity.
3. Develop a microsimulation model to evaluate health and economic impacts of policies and provide a practical decision support dashboard for policymakers for all European countries.
4. Co-develop and pilot tools to support adaptation and implementation of obesity-preventing policies in low-SEP communities.

## Methods

The OBCT project started in November 2023 and is funded for 5 years. The methodological approaches foreseen in the OBCT project are discussed per objective below:

### Knowledge

#### Objective 1: assess biological, socio-cultural and environmental obesity risks and predictors, and the role of SEP across the life course

We will work towards developing a comprehensive OBCT Risk model, in which we integrate specific biological-, socio-cultural-, and environmental obesity risk factors and their interrelationships and interactions in populations in different SEP strata, across all life stages and in life stage transitioning periods. Throughout the project, the operational definition of low socioeconomic populations will be ‘those that belong to the lowest percentiles of any socio-economic indicator (education, income, occupation) in relation to the whole population to which they belong’ [31].

Regarding **biological obesity risk**, we will focus on genetic, epigenetic, and hormonal factors. Some of such factors have been identified, e.g. Polygenic Risk Scores (PRS), the epigenetic signature (episign), and long-term cortisol levels [32–35], although these individual biological aspects have never been combined into a single risk indicator. The earlier developed and pre-tested genome-wide PRS integrates all available common variants into a single quantitative measure of inherited susceptibility and will be used in our work [32].

We will use a stepwise approach to quantify individual biological obesity risk. This entails the quantification of genetic, epigenetic, and hormonal obesity risks, integrated in a biological OBCT-risk index. Furthermore, we will investigate whether this risk varies by SEP and life stage. First, we will systematically review the scientific literature on biological determinants of obesity across the life course. Second, we will retrospectively analyse PRS, episign, and hair cortisol in relation to severity of obesity, age, and SEP. To do this, we will use data from large population-based studies such as from the Northern Finland Birth Cohorts (NFBC) and the Early Growth and Genetics Consortium (EGG). In addition, we will enrich the data of these population studies using the data biobank of the *early onset severe obesity cohort* of the Erasmus MC Obesity Center. In a final step, we will combine the indicators derived from the previous steps into an individual biological OBCT-risk index. Different indices will be developed for children and adults, as risk and risk combinations are likely to be age dependent but also generation dependent since the current adult generation of adults grew up in a less obesogenic environment, creating a different phenotype due to a different gene-environment interaction. Given that weight trajectories of individuals in different PRS deciles start to diverge in early childhood, targeted interventions may have maximal impact when implemented early in life. A limitation of the thus far identified PRS and biomarkers is that they are not derived from diverse populations. We will explore strategies to use PRS and biomarkers that are adapted for diverse populations, leveraging scores from existing or emerging datasets with greater ethnic representation, where possible.

**Figure 2.**
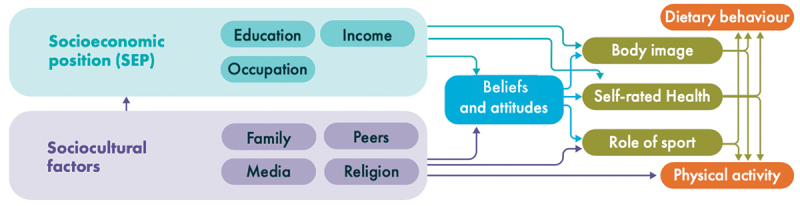
Conceptual starting framework capturing important pathways and association between socio-cultural factors and obesity-related behaviours. This diagram will act as starting model to guide meta-reviewing and the development of the socio-cultural risk index calculations to address part of Objective 1. Some arrows, denoting possible pathways, have not been included to reduce the visual complexity. Adapted from McCabe et al. 2011 [37].

Regarding **socio-cultural obesity risk**, OBCT will employ evidence reviews and de novo epidemiological aetiological analyses. The epidemiological approach will combine the analysis of individual psychological risk factors for obesity, conditioned by socio-demographic and community-level factors (age-distribution, socio-economic deprivation index, ethnic, cultural, and country of birth factors, etc.), in large administrative cohorts, and in the modelling of the direct and indirect pathways (example in Figure 2) in prospective birth cohorts (Table 1). Importantly, this framework aligns with the social determinants of health equity paradigm [36], acknowledging the role of structural factors, including structural discrimination, in shaping resource access and associated obesity risks across ethnic groups. We will review the scientific literature on sociocultural factors in obesity among low-SEP populations, focusing on community influences, family, peers, and media across the life course. Evidence will be systematically synthesized to identify patterns and gaps. Using large cohorts from Denmark and Finland (see Table 1), we will analyse how area deprivation and social factors affect weight over time, and prospective birth cohorts from Finland and the ECCN will examine pathways linking socio-cultural influences, psychology, behaviours, and BMI, stratified by sex and SEP. The methodologies employed will contribute to i) defining the relevant socio-cultural factors (and recommendation for harmonisation), ii) refining the evidence supporting their effects (and the factors of personalisation across the life course) on the risk of obesity, iii) modelling the direct and indirect pathways linking socio-cultural factors and obesity through behavioural and psychological factors, and iv) translating this model into composite scores that will define the Socio-cultural OBCT risk index.

**Table 1.** Indicative selection of cohort studies and registries considered for (secondary) analyses in the OBCT-project.

Data source	Type	N	Ages	Time period	Bio	SC	SEP	Sex	OB	Beh
US-Health Professionals Follow-Up	AC	51,529	40–75	1986-now	X	X	X	X	X	X
US-Nurses’ Health Study I	AC	121,701	30–55	1976-now	X	X	X	X	X	X
US-Nurses’ Health Study II	AC	116,429	25–42	1989-now	X	X	X	X	X	X
NL-Doetinchem cohort study	AC	7,769	20–59	1987-now	X		X	X	X	X
UK-Avon Longitudinal Study of Parents and Children	BC	14,000	0–20+	1991-now	X	X	X	X	X	X
NL-ABCD Study	BC	8,000	0–20+	2003-now			X	X	X	X
NL-Lifelines	AC	20,000	0–99	2006-now	X	X	X	X	X	X
NL-HELIUS	AC	24 789	18–70	2011-now	X	X	X	X	X	X
FI-Northern Finland Birth Cohort 1966	BC	12,068	0–55	1966-now	X		X	X	X	X
FI-Northern Finland Birth Cohort 1986	BC	9,362	0–35	1986-now	X	X	X	X	X	X
FI-Institute of Health and Welfare (THL) registers	ADM	>1M	0–34	1987–2016		X	X	X	X	
DK- Copenhagen Infant Health Nurse Records	ADM	92,902	0–64	1959-now		X	X	X	X	X
DK-Copenhagen School Health Records Register	ADM	406,350	0–93	1930-now		X	X	X	X	X
UK-SHARE	ADM	130,000	16–99	NA	X	X	X	X	X	X
NL-GECCO	AC, BC	>500,000	0–99	1968-now	X		X	X	X	X
NL-Early onset severe obesity cohort	BC	5,000	0–5	2010	X		X	X	X	
NL-TRAILS	BC	2,230	10–25	2000	X	X	X	X	X	X
NL-PIAMA	BC	3,963	0–25	1996			X	X	X	X
BR-Pelotas Birth Cohort Study	BC	4,275	0–7	2015-now	X		X	X	X	X
NO-MoBa	BC, AC	>90,000	0–99	1998–2008	X	X	X	X	X	X
UK-Biobank	AC	500,000	18–80	2012	X		X	X	X	X
EPIC-EUROPE	AC	514,487	30–99	1992	X		X	X	X	X
NO-HUNT Study	AC	230,000	13–99	1984	X		X	X	X	X
ES- HHH Cohort	AC	1,545	40–80	2017-now		X	X	X	X	X

ADM: Administrative cohort. AC: Adult cohort. BC: Birth cohort. Bio: biological. SC: socio-cultural. SEP: socio-economic position. OB: Overweight and obesity indicators: Beh: Lifestyle behaviours.

For in-depth investigations into **environmental obesity risks**, OBCT will build upon recent pioneering work in the Netherlands, where a Dutch environmental obesogenic index has been developed based on data from the Geoscience and hEalth Cohort COnsortium (GECCO) [38–40]. This index was based on an evidence-based and expert-informed theoretical framework on built environmental obesogenic characteristics such as walkability, drivability, and the healthiness of the food environment. The evidence used to create the index included a recent umbrella review on environmental determinants of obesity [41]. The Dutch preliminary index consists of 17 components related to the food and physical activity environments. Within OBCT, we will use the Dutch index as a starting point. We will calibrate and refine the index using Dutch individual BMI data as outcome (from Statistics Netherlands) and explore a refined selection and weighting of the various components in relation to the overall index score in a data-driven way. The calibrated environmental OBCT risk index will serve as an example for mapping the obesogenicity of all neighbourhoods in Europe, as described in Objective 5.

To develop an **integrated OBCT risk prediction model**, the knowledge and understanding of the biological, socio-cultural, and environmental risks associated with obesity will be compiled and reconciled in a microsimulation model. Health-GPS, a policy simulation tool based on the microsimulation model, was developed as part of the H2020 STOP project on childhood obesity [42,43] and will be used to combine the risks associated with obesity listed above. The biological, socio-cultural, and environmental risks estimates will be incorporated into the Health-GPS model. The model can simulate obesity risk for each individual in a synthetic population reflecting the characteristics of a real population, as well as broader demographic and epidemiological dynamics of NCDs in that population of interest. Health-GPS relies upon demographic data from the Human Mortality Database and UN population projections, as well as epidemiological data from the Institute for Health Metrics and Evaluation and the NCD Risk Factor Collaboration (NCD-RisC). NCD-RisC is a worldwide network of health scientists that provides rigorous, high-quality population-based, longitudinal data on risk factors for NCDs for all countries and territories in the world [1].

#### Objective 2: determine the importance of specific obesity-related behaviours to prevent obesity at key life stage transitions

We will start by performing systematic (umbrella) reviews of the associations between physical activity, sedentary behaviours, and specific dietary behaviours in the prevention of obesity during key life transition stages. These reviews will focus on physical activity intensities and sedentary time, consumption of specific foods including (ultra-) processed foods and diet-related behaviours such as eating frequency, meal skipping, snacking, and home-cooking, their effects on weight change and risk of obesity across life stages and SEP. We will mainly focus on life stages from adolescence to adulthood (age 15 to 40 years), since key life transitions occur during those life stages that affect obesity-related behaviours and provide a window of opportunity to prevent overweight and obesity. Where needed, the available evidence will be complemented with additional analyses of longitudinal cohorts covering different life transition stages. We will then identify, harmonise, and analyse longitudinal data on the associations between self-reported and device-measured physical activity, sedentary behaviour, and dietary behaviours with development of obesity during key life transition stages. Multiple cohorts with data across different life transitions stages have been identified. An initial set of relevant cohort studies and registries are listed in Table 1, along with information on the type of study, number and age range of respondents, period across the lifecourse of measurements and whether biological, sociocultural, SEP, behaviours, and weight status indicators are measured. Following the identification of the main behavioural drivers, we will assess the causality of behaviour-obesity relationships, and determine if and to what extent the direction of causality is different depending on SEP. We will disentangle the bi-directional relationships (i.e. behaviours drive obesity, but are also driven by obesity), and account for reverse causation with causal inference methods for observational data such as causal mediation analysis, trial emulation in observational data, and/or the use of counterfactual frameworks.

#### Objective 3: develop and test a holistic obesity risk screener

**Figure 3.**
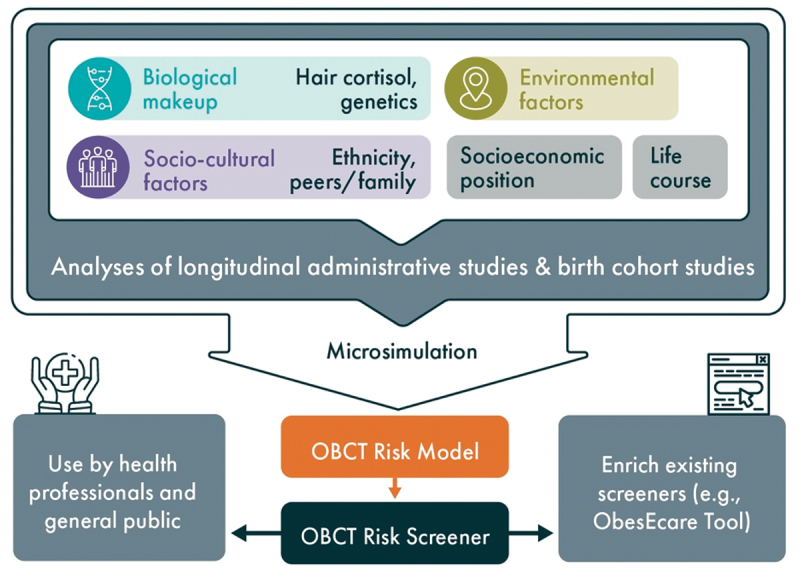
Schematic representation of the development and application of the OBCT risk screener.

The OBCT Risk Screener will be based on the biological obesity risk index, the socio-cultural obesity risk index, and the environmental OBCT risk index, and its associated OBCT risk prediction model (Objective 1). The knowledge acquired with regards to the relative importance of these risks in youth and across SEP strata is harnessed. Whereas, the indices and integrated epidemiological studies are based on explaining the causes of obesity, the risk prediction model and the screener focus on prediction. The screener is designed to assess obesity risk in children up until young adulthood to leverage the greatest obesity prevention potential. Two versions will be developed: a version for the general public and one for health professionals. The public obesity risk screener will be a practical, easy-to-administer instrument consisting of a short set of questions inquiring postal code (to determine environmental obesity risk), SEP indicators (e.g. educational level, household income, etc.), age, sex, ethnicity, and socio-cultural factors (to determine socio-cultural obesity risk), medication use, lifestyle behaviours, and discriminating questions to identify biological risk (e.g. BMI percentile, waist circumference percentile, comorbidities, etc). The more comprehensive professional application uses the public screeners as a basis but is extended with a buccal swab and a hair strand to determine PRS, episign, and cortisol levels to improve prediction. The various inputs will be weighed based on the integrated OBCT risk prediction model using microsimulation (Objective 1) and categorically scaled. The screening tool will have the form of a web-based instrument that can be applied to a diverse group, as well as to enrich existing screening instruments (Figure 3).

Initial versions of the holistic OBCT Risk Screener will be presented to the members of the European Coalition of Patients living with Obesity (ECPO) to test the feasibility of assessment and ensure non-stigmatizing, easy to understand language. Prototypes will be further pilot tested for their assessment feasibility, acceptability, and comprehension by health professionals working in communities and populations of various life stages in the Netherlands, Spain, Germany, and France. For health professionals, the prototype OBCT Risk Screener will particularly be useful in terms of: 1) empowering individuals to identify their risk and acquire knowledge and recommendations on how to take action; 2) acting as low-stakes starter for discussion with their patients/clients or their parents on the importance of maintaining/achieving a healthy body composition; 3) identifying specific domains of risk to pay special attention to; 4) tailoring advice on lifestyle changes in accordance with upcoming life course transitions; and 5) enriching other existing screeners. The professional version is made available for health professionals working in community centres such as medical doctors, dietitians, and other medical specialists.

### Maps

#### Objective 4: provide country-specific estimates of trends in general and abdominal obesity at key stages of the life course, Europe-wide

To map country-specific trends in indicators of general and abdominal obesity, we will harness the rich network and data resources of NCD-RisC [1]. The collaboration currently has data from over 3,500 population-based health surveys carried out since the 1960s and includes the anthropometric status of over 200 million participants from middle childhood to old age. We will expand the NCD-RisC data to include measures of abdominal adiposity, namely waist circumference, to calculate waist-to-height ratio. The existing data on body mass index (BMI) and data on waist-to-height ratio will be analysed to make country-specific estimates of trends in general and abdominal obesity at key stages of the life course including late childhood and adolescence (5–19 years), early adulthood (20–34 years), late adulthood (35–69 years), and late adulthood (70+), for all countries in Europe and European regions. Given the interest in trends at different stages of the life-course, the meta-regression model used for pooling and analysing data will be modified to allow trends to vary relative to age. We will also explore the potential of harmonising SEP indicators, to analyse differential trends across SEP groups. We will investigate the degree to which BMI is correlated with the more health-relevant waist-to-height ratio across Europe and across age groups [44,45], and how the two metrics have changed in relation to one another.

#### Objective 5: quantify obesogenic neighbourhood characteristics of all local administrative units within Europe

To address this objective, we will take the evidence- and theory-based environmental OBCT risk index developed for Objective 1 as a starting point. To characterise obesogenicity of all local administrative units of Europe in a standardised way is challenging due to uncertain and uneven availability of the same data. We will benefit here from the findings of H2020 projects such as Expanse [46], for which a start was made to assess neighbourhoods in terms of their food environment [47] and walkability. Furthermore, we will use our extensive experience within the FP7 SPOTLIGHT project [48], in which we gathered and operationalised cross-European data on environmental obesogenic characteristics [49–51]. We will also web-scrape Places of Interest (POIs). Objectively measured data will be prioritised over self-reported data [52]. The centralised and operationalised componential data will be combined into a full European Environmental OBCT risk score. The ten-step procedure recommended in the OECD Handbook on Constructing Composite Indicators will be followed for this purpose [53]. The comprehensive and high-resolution characterisation of all areas will feed into an interactive web-atlas. This atlas will be made available to the public, allowing people to obtain information on obesogenic neighbourhood characteristics specific to all European local areas, municipalities, regions, countries, and the whole of Europe.

The index enables the examination of interactions between genetic and socio-cultural obesity risks and studying the associations with further downstream health outcomes such as hypertension, cardiovascular disease mortality, and morbidity. Furthermore, the obesogenic neighbourhood atlas allows for the measurement, monitoring, and benchmarking of neighbourhoods in terms of their potential influence on obesity. Consequently, it will aid municipal and regional policymakers in directing their preventive efforts in specific neighbourhoods in need of infrastructural changes to encourage healthy lifestyles and reduce the disease burden.

#### Objective 6: characterise obesity-related cardio-metabolic risk profiles by gender, age, SEP within representative European countries

We will characterise obesity-related cardio-metabolic risk profiles of those with obesity, and their variation within and between representative European countries. Some studies have used concepts such as metabolic syndrome and metabolically healthy obesity to identify groups of people with specific pre-determined risk-factor profiles [54–57]. However, few studies, especially population-based studies, thus far has provided a data-driven and comprehensive characterisation of all cardio-metabolic risk-factor profiles in a population. We will take a data driven approach to identify a comprehensive set of cardio-metabolic risk profiles among those with and without obesity, stratified by age group and gender [57]. The variation in obesity-related cardio-metabolic risk profiles is characterised within and between a selection of representative European countries, and examined to uncover and demonstrate the requirements and opportunities for prevention and management of cardio-metabolic health risks associated with obesity in entire populations and population subgroups. Data will be gathered from an extension of the NCD-RisC within Europe to bring together, and harmonise, data on multiple risk factors. We employ statistical machine learning methods to identify cardio-metabolic risk profiles; defined as parsimonious and clinical/epidemiological sets of clusters to which people are assigned. Finite and infinite mixture models and latent class models will be considered.

### Tools

#### Objective 7: develop recommendations for dietary, physical activity, and sedentary behaviours tailored to key life stages and socio-economic backgrounds

Current recommendations for the prevention and management of obesity include only a few behavioural recommendations on meal skipping and snacking [58]. We will develop recommendations on specific physical activity, sedentary time, and dietary behaviours tailored to key life stages, SEP, and important drivers of obesity risk. These recommendations will be based on knowledge gained on the relative contributions of different drivers of obesity risk (biological, socio-cultural and environmental), the OBCT screener to assess these risks, and key physical activity and dietary behaviours contributing to obesity risk at different life stages obtained from Objective 2. Current recommendations for the general public or for the management of obesity include generic advice on dietary behaviour and physical activity according to age group [59]. Most dietary recommendations for the general public are focused on the reduction of NCD risk and are not specifically designed to prevent obesity. Moreover, although a few dietary guidelines also focus on diet-related behaviours such as meal frequency or home-cooking [60], most guidelines do not include these behaviours [61]. We will review and grade the evidence of behavioural specific recommendations for obesity prevention according to Grading of Recommendations, Assessment, Development and Evaluations (GRADE) [62]. Subsequently, we will provide additional recommendations to be incorporated in a series of guidance documents and potential add-ons to current diet and physical activity guidelines. The recommendations will be established with a committee consisting of members with different relevant expertise (dietitians, behavioural scientists, medical doctors etc.) in a series of three two-day meetings, to evaluate the review and grading of the evidence. This will be done with support of the European Association for the Study of Obesity (EASO)’s health professional education and using national obesity networks. These networks are affiliated with OBCT partners EASO and World Obesity Federation (WOF) and gather in project-led and external events/conferences aimed at healthcare professional organisations and their national governing bodies. These recommendations will be disseminated to key stakeholders that work with the target groups, including community health workers, dieticians, nutritionists, and medical doctors.

#### Objective 8: determine the potential impact of obesity-related policies on SEP-related inequalities in obesity

As recognised by the WHO, there is a need to monitor and evaluate the impact of efforts to reduce obesity [25]. We will conduct two systematic reviews of literature analysing policies that were implemented in communities that may be characterised by lower SEP. Agentic polices, such as raising awareness about healthy diet/physical activity guidelines, demand individual agency (i.e. relying on an individual’s knowledge, health literacy, engagement, ability, and power to act). Such policies may in fact increase inequalities in obesity, as populations with fewer resources and more challenges are less likely to benefit from them. In contrast, structural policies eliminate or restrict choice and put limited demands on individual resources [63,64]. Structural policies include banning the advertising or sale of unhealthy food in certain places, meaning that people are, therefore, less exposed to unhealthy options. Agento-structural polices include changes to the structural environment, but also for individual agency [63]. An example is the urban design to facilitate walking and cycling. We will focus on structural and agento-structural policies in terms of their 1) effects on weight-related and behavioural outcomes, as well as implementation outcomes, such as feasibility or acceptability by relevant stakeholders; and 2) policy implementation strategies used in polices applying the whole-of-community approach to deliver obesity prevention. In sum, the reviews will focus on these characteristics of policies that are likely to be favoured by the relevant stakeholders due to their implementability, promote multisectoral collaboration, and prompt the whole-of-community system changes in low-SEP communities. The results will feed directly into the co-development of the OBCT tools for implementation of obesity preventing policies.

#### Objective 9: develop a microsimulation model to evaluate health and economic impacts of policies and provide a practical decision support dashboard for policymakers for all European countries

We will expand the Health-GPS policy microsimulation tool used in Objective 1 to explore the economic and health impacts of obesity-related policies across various European countries. Health-GPS relies on complex datasets as inputs to inform country-specific demographic trends, along with individual exposures to modifiable and non-modifiable risk factors associated with obesity and other related NCDs. Using these data and simulations of populations over time, Health-GPS estimates the health and economic impacts of policies aimed at reducing exposure to given (e.g. dietary) risk factors, and the resulting influence on the incidence and outcomes of NCDs. Policy scenarios, involving a specific policy design and estimates of intervention impacts, will be developed for national-level policy recommendations taking equity into consideration as well as risk of misuse by decision-makers. Each policy scenario will be simulated in comparison to a baseline, to estimate the expected impacts of interventions. Country-specific simulated results will be displayed on an interactive dashboard, allowing policymakers, other relevant stakeholders, and the public to explore the impact of policy scenarios in relevant settings and under alternative sets of assumptions.

#### Objective 10: Co-develop and pilot tools to support adaptation and implementation of obesity-preventing policies in low-SEP communities.

OBCT will attempt to make existing relevant public and regional policies practicable for those working with the target population; in particular, those in low-SEP communities. A stepwise approach will be followed: 1) Tools will be co-developed based on theoretical frameworks for implementation [65] and whole-of-community approaches [66,67], taking into account ethical and regulatory constraints. European and national stakeholders and experts will be consulted in a series of workshops, drawing on participatory and co-creating methods [68,69] with the aim to facilitate the adaptation and implementation of national policy recommendations for obesity prevention into actions in low-SEP communities; 2) Pilot test the tools in low-SEP communities/municipalities in Norway, Poland, and Spain using a quasi-experimental design to evaluate community readiness for obesity action and a thorough mixed-methods implementation evaluation; 3) The tools will be refined based on the outcomes of the pilot; and 4) the tools will be provided to relevant stakeholders/public authorities in the communities. The tools will form part of the extensive training portfolio provided by EASO, which includes courses, masterclasses, e-learning, and uptake in guidelines. The tools will also be added to WOF’s Policy Dossiers, as well as disseminated among European and national stakeholders and experts in the workshops conducted in the development phase.

## Discussion

OBCT will provide insights into the interaction of relevant obesity risk factors in different domains, develop a comprehensive obesity risk screener, and deliver tools to effectively adapt and implement obesity-preventing policies, with a focus on low-SEP communities. This latter is especially important given the widening health disparities across SEP strata, which may be partly attributed to obesity-related factors. The foreseen methodologies in OBCT are effective to address this issue by elucidating the mechanisms through which SEP disparities influence health outcomes, particularly in the context of obesity. In addition, they will increase the needed knowledge on how various biological, socio-cultural, and environmental risk factors interact with one another across the life course and which approaches may affect the critical risk factors with the aim of preventing obesity. The life-course approach is important as there may be sensitive periods in which the effect of certain risk factors is magnified as compared to the same risk factor exposure in another time period. Moreover, the importance and interplay of the variety of risk factors likely change across the life course and might be specific to SEP background. This holistic analysis should be focused not only on the individual but also on their family and the wider community in which the individual resides. Communities may share a sense of place in a given geographical area or setting (e.g. a neighbourhood, school, workplace) and are typically exposed to shared policies as well as structural determinants. It has even been posited that a postal code may be a better predictor of health outcomes than a genetic code [70]. Yet, to better understand the drivers of obesity, we need to combine both these codes, as well as complement them with socio-cultural contextual information, as we intend to do in OBCT. To reduce inequalities in obesity, improved knowledge of the combination of these cumulative and interactive risks over the life course in low-SEP communities is essential.

## Conclusions

OBCT’s outputs will determine where obesity risk is greatest, and in which domains obesity is to be targeted. They will empower the research community, policymakers, health professionals, and residents in Europe to adapt and implement knowledge and strategies to effectively reduce obesity risk, thereby helping to prevent obesity – particularly in low-SEP communities.

## List of abbreviations

**Table:** 

BMI	-	Body mass index
EASO	-	European Association for the Study of Obesity
ECCN	-	European Child Cohort Network
ECPO	-	European Coalition for People living with Obesity
EGG	-	Early Growth and Genetics Consortium
GECCO	-	Geoscience and health cohort consortium
NCD-RisC	-	Non-Communicable Disease Risk Factor Collaboration
NCDs	-	Non-communicable diseases
NFBC	-	Northern Finland Birth Cohorts
OBCT	-	Obesity: Biological, socio-Cultural, and environmental risk Trajectories
OECD	-	Organisation for Economic Co-operation and Development
PRS	-	Polygenic risk score
SEP	-	Socio-economic position
WOF	-	World Obesity Federation

## Data Availability

This manuscript does not contain any data. Research data used or generated over the course of the project will be open for access under the principle ‘as open as possible, as closed as necessary’, in adherence to the conditions required by the grant agreement and internal academic organisations of involved OBCT partners.
